# Postural control is destabilized by theta-burst stimulation over the DLPFC during the performance of a concurrent working memory task

**DOI:** 10.1007/s00221-025-07150-4

**Published:** 2025-10-08

**Authors:** Sam Carey, Ramesh Balasubramaniam

**Affiliations:** Cognitive & Information Sciences, Sensorimotor Neuroscience Laboratory, University of California, 5200 N Lake Road, Merced, CA 95343 USA

**Keywords:** Postural sway, Transcranial magnetic stimulation, Cognitive load, N-back, Working memory, DLPFC

## Abstract

Human balance control requires the coordination of the human motor, cognitive, and sensory systems. Several studies have assessed the interaction between postural control and cognitive tasks when performed concurrently, but this interaction is yet to be fully understood. The current study aims to examine whether the postural control system is impacted by working memory, but more specifically by downregulating the Dorsal Lateral Prefrontal Cortex (DLPFC) during the performance of a working memory task using repetitive Transcranial magnetic stimulation (TMS). Additionally, this study aims to understand if there is a lateralization effect during TMS of the left vs right DLPFC, an area that is activated during the performance of working memory tasks. Postural sway of healthy young adults was recorded while participants performed a modified working memory task of varying difficulties across 3 blocks. The sessions were randomized to include continuous theta-burst transcranial magnetic stimulation of the left DLPFC, right DLPFC, or a sham condition. Our results showed that the performance of a cognitive task caused a destabilizing effect to occur in the postural control system, and with the introduction of TMS to the DLPFC, this effect was intensified. There was no difference between the left vs right stimulation of the DLPFC, but the overall effect seen shows that the cognitive and postural control systems rely on similar mechanisms that, when performed simultaneously, cause a negative interference effect.

## Introduction

Interest in postural control research dates back to the early 1900’s, ever since Sherrington’s original work in 1906, there has been a need to research postural control as its own independent system as well as its interaction with human behaviors through a dynamical systems approach. Stability, within the postural control context, refers to the ability of an individual to maintain or return to a desired position or orientation in space, particularly during upright stance. It reflects the capacity of the sensorimotor system to minimize excessive movement and maintain balance by regulating body movement within a stable base of support. Maintaining postural control and stability is essential for successfully performing everyday tasks. Rather than being an end in itself, postural control serves to support a wide range of goal-directed behaviors across the human lifespan (Riccio & Stoffregen 1988). Due to this perspective, posture is often considered an automatic task, requiring little attentional resources to maintain stability while standing (Schneider & Fisk 1983). The concept of automaticity applies to postural stability, where rapid corrective actions are crucial for countering destabilizing effects (Pruszynski 2014) and these adjustments occur without the need for conscious focus on posture, as doing so could interfere with the performance of other tasks.

Despite its seemingly simple nature, the complexity of the postural control system is often underestimated. Historically, researchers have discussed the super-ordinate organization of subsequent task completion in reference to postural control (Stoffregen et al. 1999), neglecting the influence postural control has on the task or vice versa. This type of separation between the postural control system and other behaviors remains common unless that interaction is intentionally under the lens of study, such as in dual-tasking paradigms. However, more recent research has shed light on the sensitivity of the postural control system to both cognitive influences (Andersson et al. 2002; Abou Khalil et al. 2020; Carey and Balasubramaniam 2025) and altered sensory feedback (Yeh et al. 2014; Ross & Balsubramaniam, 2015; Ross, McGann, & Balasubramaniam, 2016a; Carey et al. 2023; Carey et al. 2024). This work shows how postural control, while seemingly automatic in nature, is easily altered given the external and internal environment. Further extending the idea that postural sway, and human behavior as a whole, is a dynamical system and deserves to be studied as such.

During dual-tasking, it is often thought that one of the tasks becomes more ‘automatized’ so the concurrent task can be performed with little to no interference. Tasks such as walking and talking, writing while listening to music, or standing while reading require attention to be divided amongst the tasks being performed, and often result in the performance of one or both of the tasks to change (Shumway-Cook et al. 1997; Prado et al. 2007; Ross et al. 2011; Olivier et al. 2010). While balance is typically considered an automatic or ‘subconscious’ task, multiple studies have suggested that attentional resources are still needed to maintain balance during increased dual-tasking behaviors, which varies according to environmental constraints and internal limitations (Woollacott and Shumway-Cook 2002; Maylor & Wing 1996; Pellecchia 2003), and recent work has shown how performing a cognitive task while standing negatively impacts postural dynamics (Carey and Balasubramaniam 2025).

Historically, the theories often used to explain this interference effect (single-channel, capacity-sharing, unitary-resource capacity, or the bottleneck theory) are attributed to a competition between attentional resources, which would cause a decrease in performance of one, or both, of the tasks being executed (Brown et al. 1999). However, if it were an attentional control or capacity issue, that would posit that regardless of what tasks are being performed, if dual-tasking were to occur, performance would always decline. Yet, recent work has shown that this is not always the case, and given the situation of the task, the performance varies.

Multiple studies have found decreases in postural sway amplitude (Andersson et al. 2002), while others, particularly those involving clinical populations, have observed increases in postural sway amplitude or variability (Pellecchia 2003; Redfern et al. 2001). Ramenzoni et al. (2007) found that postural sway was influenced by specific components of cognitive tasks rather than the task as a whole. They reported increased sway variability during the encoding phase, but decreased variability during the rehearsal phase in the anterior–posterior and medial–lateral directions of sway. They also observed that the cognitive task's effects varied between visual and verbal stimuli. Similarly, Maylor and Wing (1996) found that older adults were more susceptible to the effects of cognitive load than younger adults. In a digit recall task involving spatial memory, older adults exhibited increased postural sway whereas younger adults showed reduced sway relative to baseline conditions. In contrast, tasks like random-digit generation, silent counting, and arithmetic counting showed no age-related interaction. Additionally, research by Maylor, Allison, and Wing (2001) indicated that postural sway decreases during memory encoding and increases during retrieval for both spatial and non-spatial tasks.

These discrepancies in the motor performance of dual-tasking leads us to approach this problem through a different lens. Instead of viewing the problem as one of attentional constraints, new theories have arisen that reframe dual-tasking interference as one of cortical interference. Since these original theories have been posited, the idea of neural remapping theory has been introduced to better explain the differential effects seen between cognitive task completion and postural control. This theory states that through evolutionary means, our neural circuits, which once may have been singular in their activations and usage, have since evolved to be utilized for multiple behaviors as the world around us has increased in complexity. This ‘remapping’ of the neural circuitry has since caused varying behaviors to become reliant on neural regions that may overlap or even be the same circuits, and when two behaviors are performed that rely on the same, or overlapping, set of neural circuits, the performance of those behaviors declines.

The dorsolateral prefrontal cortex (DLPFC) has traditionally been identified as a key region involved in domain general executive functions. These include high-level cognitive processes such as task switching, task-set reconfiguration, interference control, inhibition, and planning. Among these, one of the most critical for the present work is its central role in working memory (Badre and Wagner 2004; Hart et al. 2013; Brunoni and Vangerhasselt 2014). Working memory, or the ability to temporarily hold and manipulate information, relies heavily on DLPFC activation (Curtis and D’Esposito 2003). Importantly, this function appears to be lateralized within the DLPFC based on the type of information being processed. The left DLPFC is predominantly involved in handling verbally and linguistically encoded information, such as language-based content, while the right DLPFC is more engaged during tasks requiring nonverbal processing, particularly those involving visual or spatial representations (Baddeley 2003; Buchsbaum and D’Esposito 2008, 2019). This hemispheric specialization underscores the flexibility of the DLPFC in supporting a broad range of cognitive demands depending on contextual task requirements.

This study aims to better understand this possibility of cortical interference between postural control and cognition. By introducing a cognitive working memory task during upright standing, this study is designed to better understand how cognition may impact stability, and similarly, how postural control may impact cognitive performance. Within this study we manipulated the difficulty of the cognitive task itself as well introducing transcranial magnetic stimulation (TMS) to the DLPFC to manipulate the participants capability of performing the cognitive task. More specifically, by downregulating the DLPFC during the performance of the working memory task using Transcranial magnetic stimulation (TMS) we will be able to better understand if the working memory systems are directly involved in the interference commonly seen between cognition and postural control. Furthermore, by applying TMS stimulation to both the left and right DLPFC we hope to better understand if there is any lateralization effect of working memory regions and their influence on postural control given the design of our task.

## Methods

### Participants

Twenty-eight healthy young adults, 15 female and 13 males, (mean age = 22.41 ± 5.83) of varying heights (66.45 ± 5.13 inches) and weights (155.01 ± 30.22 lbs.) were recruited from the undergraduate and graduate student population at the University of California, Merced. Eligibility was determined via self-reported screening measures, which excluded individuals with hearing impairments, arthritis, orthopedic conditions, or neurological disorders (Carey et al. 2023, 2024; Ross & Balasubramaniam 2015; Ross et al. 2016a, 2016b). No participants reported recent injuries or musculoskeletal disorders, and all were capable of standing unassisted for the full duration of the experimental session. The study protocol adhered to the ethical guidelines outlined in the Declaration of Helsinki, received approval from the UC Merced Institutional Review Board (IRB), and all participants provided written informed consent prior to participation.

### Protocol

Participants were instructed to stand on a force platform in a relaxed, comfortable position with their arms resting at their sides, feet shoulder width apart, and wearing headphones. To ensure consistency in base of support across trials and following breaks, a box was taped around the external perimeter of each participant's feet upon their initial placement on the force plate. Participants were given periodic breaks throughout the task, typically after every 5 to 7 trials, allowing them to sit and rest their legs. Before resuming the next block of trials, participants were asked to return to the marked stance, aligning their feet within the taped boundary and placing their arms back at their sides.

Each participant completed 15 trials in total, consisting of five trials per condition: No Cognitive Task, Easy Cognitive Task, and Hard Cognitive Task. The Easy and Hard conditions were administered in blocked formats (five trials each), while the No Task condition was distributed across three blocks: two trials at the beginning, one in the middle, and two at the end of the session. This arrangement was designed to establish a baseline measure while mitigating potential fatigue effects over the course of the experiment. To minimize confusion between task rules and reduce cognitive interference, trials of the same difficulty level were presented in blocks rather than interleaved. This blocked design ensured that participants remained consistently engaged with a single task rule per block, thereby maintaining task integrity and reducing potential task-switching costs. Each participant completed multiple blocks of each difficulty level across the study.

Participants repeated this protocol 3 times with a single session each day. The sessions were randomized to include continuous theta-burst transcranial magnetic stimulation of the left DLPFC, right DLPFC, or a sham condition. The stimulation occurred before the cognitive task completion but after mapping the participants foot box on the force plates.

Center of Pressure (CoP) was sampled at 200 Hz with an AMTI Force and Motion platform (Optima BP400600-2000 GEN 5; AMTI Force & Motion, Watertown, MA, USA). All data was collected in a single session.

### Transcranial magnetic stimulation

This study utilized a continuous theta burst stimulation (cTBS) protocol – a form of repetitive transcranial magnetic stimulation (rTMS) – to transiently downregulate activity in targeted cortical regions for approximately 20 to 40 min post-stimulation, employing the Magstim Rapid2 system. Stimulation was delivered in bursts of three pulses at 50 Hz, repeated at 200 ms intervals, for a total of 600 pulses over 40 s at 80% power of each participant’s active motor threshold (AMT) (Huang et al. 2005). In cases where a participant’s AMT exceeded the equipment’s safe operating threshold, participants were not included in the study or received stimulation.

To determine each participant’s active motor threshold (AMT), single pulse TMS was administered to the left primary motor cortex hotspot at varying intensities until visible muscle twitches were elicited in the flexed first dorsal interosseous (FDI) muscle in 5 out of 10 trials. This criterion was chosen within the lab to ensure a strong enough activation power of the stimulation without risking stimulating at too high of a power as to risk the safety of the participants. The presence of visible twitches were verified through motor-evoked potentials (MEPs) of at least 50 microvolts from the right FDI muscle. Motor hotspot location was optimized by comparing MEP size and consistency across multiple motor cortex regions.

Surface electromyography (EMG) was recorded using Ag/AgCl sintered electrodes placed over the belly of the right first dorsal interosseous (FDI) muscle. A ground electrode was positioned over a bony prominence distal to the right elbow to ensure signal stability. Single pulse transcranial magnetic stimulation (TMS) was administered to the left primary motor cortex using a figure-of-eight coil (Magstim D702, 70 mm coil, Carmarthenshire, United Kingdom). The coil was held tangentially to the scalp at a 45-degree angle relative to the anterior–posterior midline, in accordance with established protocols.

Magstim Visor2 3-D motion capture-guided neuronavigation system was employed to scale each individual participant’s brain model to the Talairach brain using head size and shape and to guide stimulation of the Left and Right dorsolateral prefrontal cortex (DLPFC). We used 3-D coordinates determined from previous literature, target coordinates for stimulation were selected with Talairach coordinates set to R: 40, 32, 30 and L: -40, 32, 30. During cTBS, the coil was positioned tangentially to the scalp and angled at 45 degrees from the anterior–posterior midline, following the methodology described by Janssen et al., 2015. Sham cTBS was delivered over the left primary motor cortex (M1), with the coil oriented away from the participant’s head to prevent stimulation. All experimental sessions adhered to the UC Merced IRB protocol for TMS research, with a minimum of 24-h interval separating each session.

### Cognitive task

While standing, participants completed a modified N-back task featuring two levels of difficulty: Easy and Hard. Each trial lasted 90 s, with a total of 10 trials per difficulty level. The task was presented on a projector screen positioned 210 cm in front of each participant, with the screen’s center adjusted to align with each individual’s eye level. No participants reported difficulty viewing or reading the letters during the task performance.

**Fig. 1:**
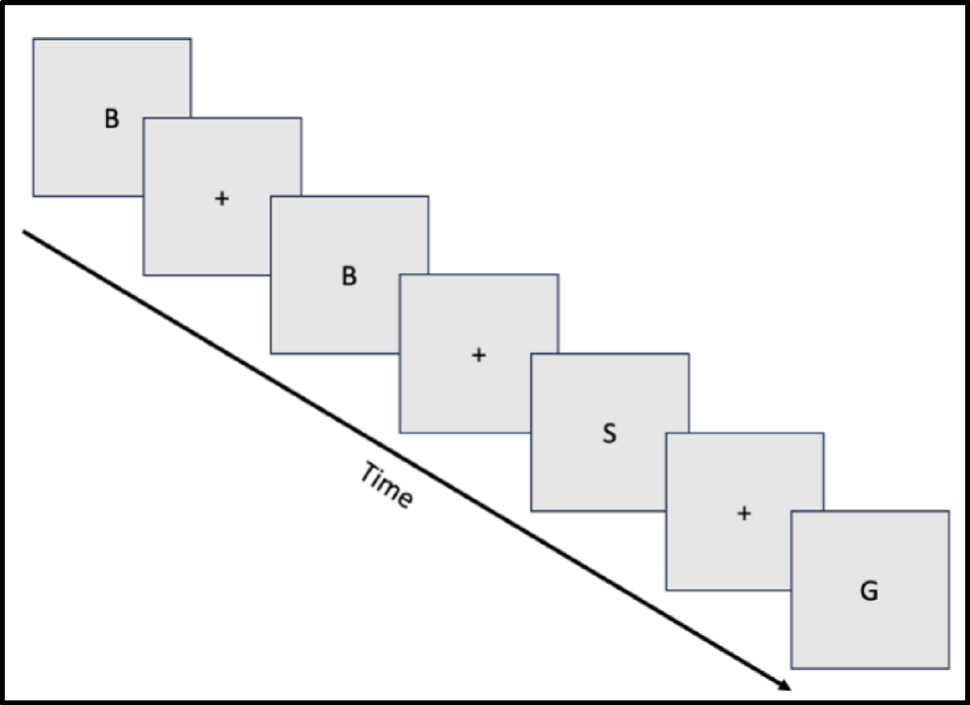
Simplified example format of the presentation of the modified n-back task

Capitalized letters appeared on the screen in a random order, with a fixation cross appearing between each letter (Fig. 1). Each letter appeared on the screen for 0.5 s, followed by a fixation cross displayed for 2.0 s, creating a repeating cycle that continued for the full duration of each trial. Every trial began and ended with a fixation cross, resulting in the presentation of 34 letters and 35 fixation crosses per trial. The letter “X” was excluded due to its similarity to the fixation cross “ + ,” while all other letters within the alphabet were included.

The objective of the **EASY** condition was to count the number of instances in which any letter was repeated following a single fixation cross. For example, if the sequence of letters presented was: *A* + *A* + *F* + *K* + *R* + *R* + *T* + *P*, participants would report a total count of 2, once for the letter “A” and once for the letter “R.” At the end of each trial, participants were asked to self-report the total count of repeated letters they observed. For the **HARD** condition, the presentation format remained the same; however, the task required a higher level of working memory, following an N-back-2 structure. In this version, repetitions were only counted if a letter reappeared with a single intervening letter and two fixation crosses between them. For instance, given the sequence: *A* + *R* + *A* + *K* + *T* + *R* + *T* + *P* + *F* + *P*, the correct response would be 3, corresponding to the valid repetitions of “A”, “T”, and “P” that adhered to the N-2 rule.

At the conclusion of each session, absolute error was calculated as the difference between the expected number of repetitions and the participant’s self-reported count across all trials within the *Easy* and *Hard* conditions. To minimize the potential for cognitive offloading, participants were explicitly instructed not to use their hands or fingers to track repetitions during task performance.

### Analyses

All CoP data was analyzed using custom scripts developed in MATLAB (MathWorks, Natick, MA, USA). To account for potential startle responses at stimulus onset, the initial 4 s of each trial were excluded from analysis. Radial sway (RS) of the CoP was calculated for each time step (i) by using the anterior–posterior (A–P; x) and medial–lateral (M–L; y) components of sway, following the methods described by Lafond et al. (2004):

$${RS}_{i}= \sqrt{{x}_{i}^{2}+ {y}_{i}^{2}}$$

Average RS was calculated for each trial and served as the primary measure of bidirectional variability in CoP during standing (Lafond et al. 2004). Although several other metrics, such as mean velocity, median power–frequency, root mean square distance, and sway area, are widely employed in the study of postural sway (Lin et al. 2008), RS provides a multidirectional account of sway dynamics. While RS is not a direct metric of stability, it captures the variability in CoP across both anterior–posterior and medial–lateral axes, offering a more nuanced depiction of sway behavior compared to the unidirectional measures like standard deviation of CoP magnitude or velocity (Lafond et al. 2004). Trial outliers were determined as trials with averages of ± 2 standard deviations from that participant’s mean within a given condition and were excluded from subsequent analyses. On average, this process resulted in the removal of 8% of the total trials (101 out of the total 1260 trials). No subject had more than 3 trials (out of 10 per condition) excluded.

Subsequently, the analysis was then repeated on RS data filtered into high- and low-frequency components to examine changes across different timescales of postural control, in accordance with the procedures outlined by Yeh et al., (2010, 2014). Low- and high-pass Butterworth filters were applied to isolate frequency bands, as in Yeh et al. 2014, with a cutoff of 0.3 Hz to distinguish between sway driven by sensory feedback and sway reflecting more spontaneous or exploratory movement, as recommended by van den Heuvel et al. (2009).

Finally, detrended fluctuation analysis (DFA) was employed to quantify the temporal dynamics of postural sway (Delignières et al. 2003; Collins and Luca 1994). DFA is a method designed to evaluate the temporal structure of time series data (in this case, CoP). Origionally introduced by Peng et al. (1994), DFA is a form of scaling analysis method that produces a scaling exponent (α), which offers information concerning the correlational properties of the CoP signal. When the DFA exponent falls within the range 1 < α < 1.5, postural sway is considered to exhibit antipersistent dynamics. In this context, antipersistence implies that sway movements occur in successive steps, semi-random steps that are not biased in a consistent direction, indicative of a healthy and adaptive postural control system. The DFA procedure was implemented following the methods outlines by Blázquez et al. (2010), using the same analytical parameters. For a comprehensive explanation of the DFA technique and its applications within postural control research, see Blázquez et al. (2010) and Delignières et al. (2003).

All statistical analyses were conducted using R (Version 1.3.1093). Linear mixed-effects (LME) regression models were fitted using the **lme4** package (Bates et al. 2015). To examine RS, LME models were constructed to account for variance attributable to both experimental condition and participant. Corrections for multiple comparisons were calculated using the Tukey’s method. Estimated marginal means and pairwise comparisons, along with associated confidence intervals, were extracted from the LME models using the emmeans R package (Lenth 2022). Multiple model variations were compared using likelihood ratio tests and information criteria, resulting in the current model being chosen based on its superior model fit and parsimony.

To analyze the effects of stimulation and cognitive task condition on postural sway, we fit a linear mixed-effects model using the lmer function from the lme4 package in R. The model included fixed effects for stimulation condition (Stim) and cognitive task condition (Condition), and a random effects structure allowing both intercepts and slopes for trial number to vary by subject (1 + Trial | Subject). This structure accounts for individual differences in baseline sway and learning or fatigue-related changes over trials. The outcome variable was radial sway, which reflects the magnitude of postural movement.

A power analysis was conducted to determine the minimum number of participants required to detect a significant effect of cognitive task difficulty and stimulation condition on radial sway. Based on a large effect size (Cohen’s f > 0.4) and the study design. featuring two cognitive difficulty levels (easy, hard) and three stimulation conditions (Left DLPFC, Right DLPFC, and Sham), a linear mixed effects model was assumed to account for within-subject variability. The analysis indicated that approximately 30 participants would be sufficient to achieve 80% power at an alpha level of 0.05 to detect a significant main effect or interaction.

## Results

### Cognitive performance

A linear mixed-effects model was conducted to examine the effects of transcranial magnetic stimulation (TMS) to the dorsolateral prefrontal cortex (DLPFC) and cognitive task difficulty on task performance. The model included fixed effects for TMS to the right DLPFC (TMSRight), TMS to the left DLPFC (TMSLeft), Sham TMS, and cognitive task difficulty (with Hard coded as a contrast against the baseline condition).

**Fig. 2:**
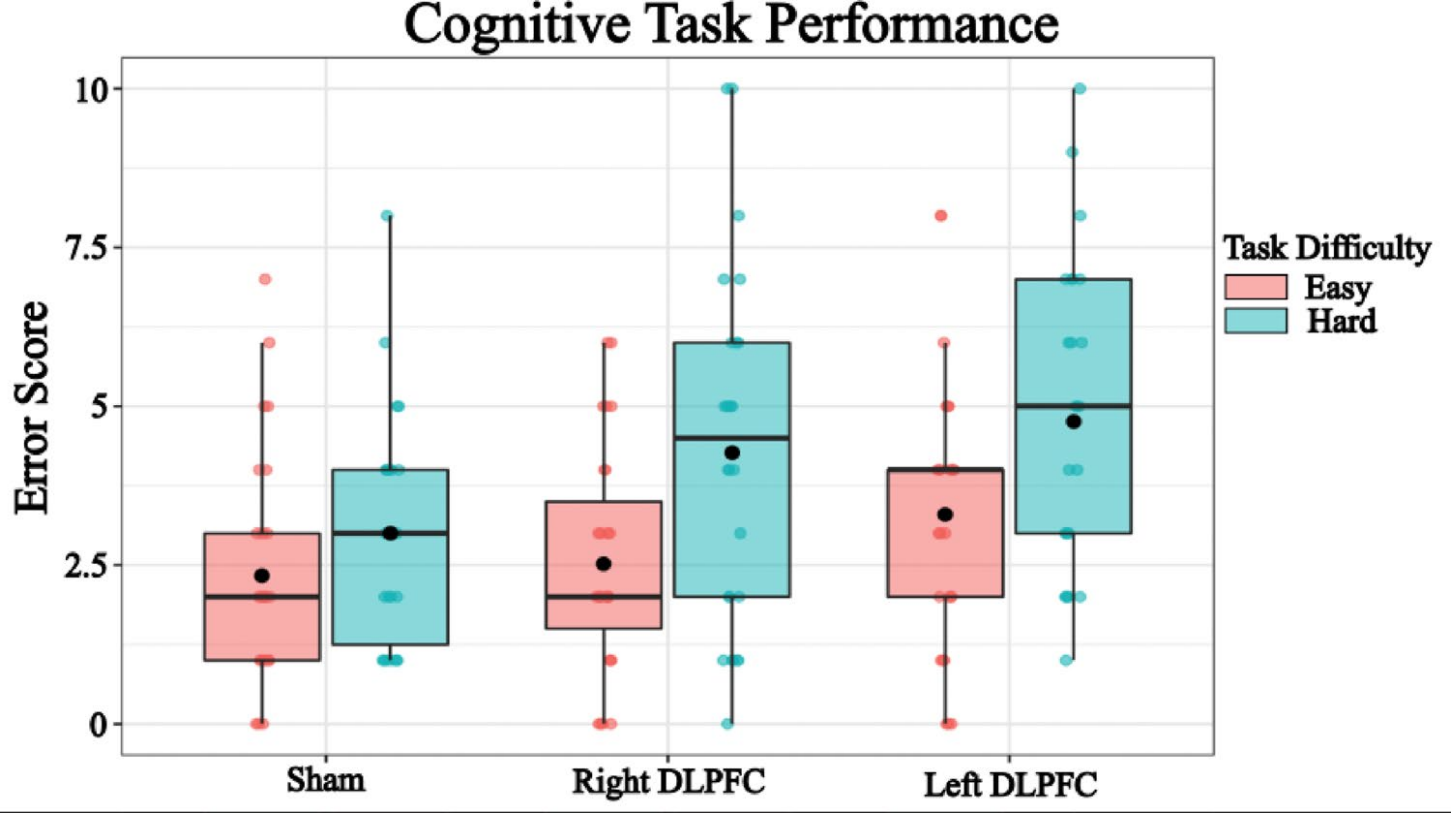
Cognitive Performance. Individual points represent average error score for each participant per condition. Error scores equate to the number of absolute errors made on the task per trial. Box and whiskers plot with the solid black line representing the median, the solid black dot representing the mean, and the extending lines showing the maximum and minimum values

The model revealed a significant intercept, estimate = 1.95, SE = 0.46, t = 4.26, *p* < 0.001, indicating the estimated error score in the baseline condition without TMS and under the easier cognitive task. Stimulation to the right DLPFC was associated with a marginally significant increase in error score (estimate = 0.72, SE = 0.37, t = 1.95, *p* = 0.051), while stimulation to the left DLPFC resulted in a significant increase in error score (estimate = 1.57, SE = 0.37, t = 4.24, *p* < 0.001). These results suggest that TMS to the left DLPFC has a stronger effect on task performance compared to the right DLPFC (Fig. 2).

Additionally, cognitive task difficulty significantly decreased performance. Performing the hard cognitive task led to a significant increase in error score compared to the easier task (estimate = 1.77, SE = 0.30, t = 5.83, *p* < 0.001), indicating that increased cognitive demand negatively affects balance.

Overall, these findings suggest that both TMS to the DLPFC and cognitive task difficulty impair cognitive performance, with left DLPFC stimulation having a greater impact. This supports the notion that the prefrontal cortex plays a crucial role in working memory performance, or cognitive performance more broadly speaking.

### Radial sway

**Fig. 3:**
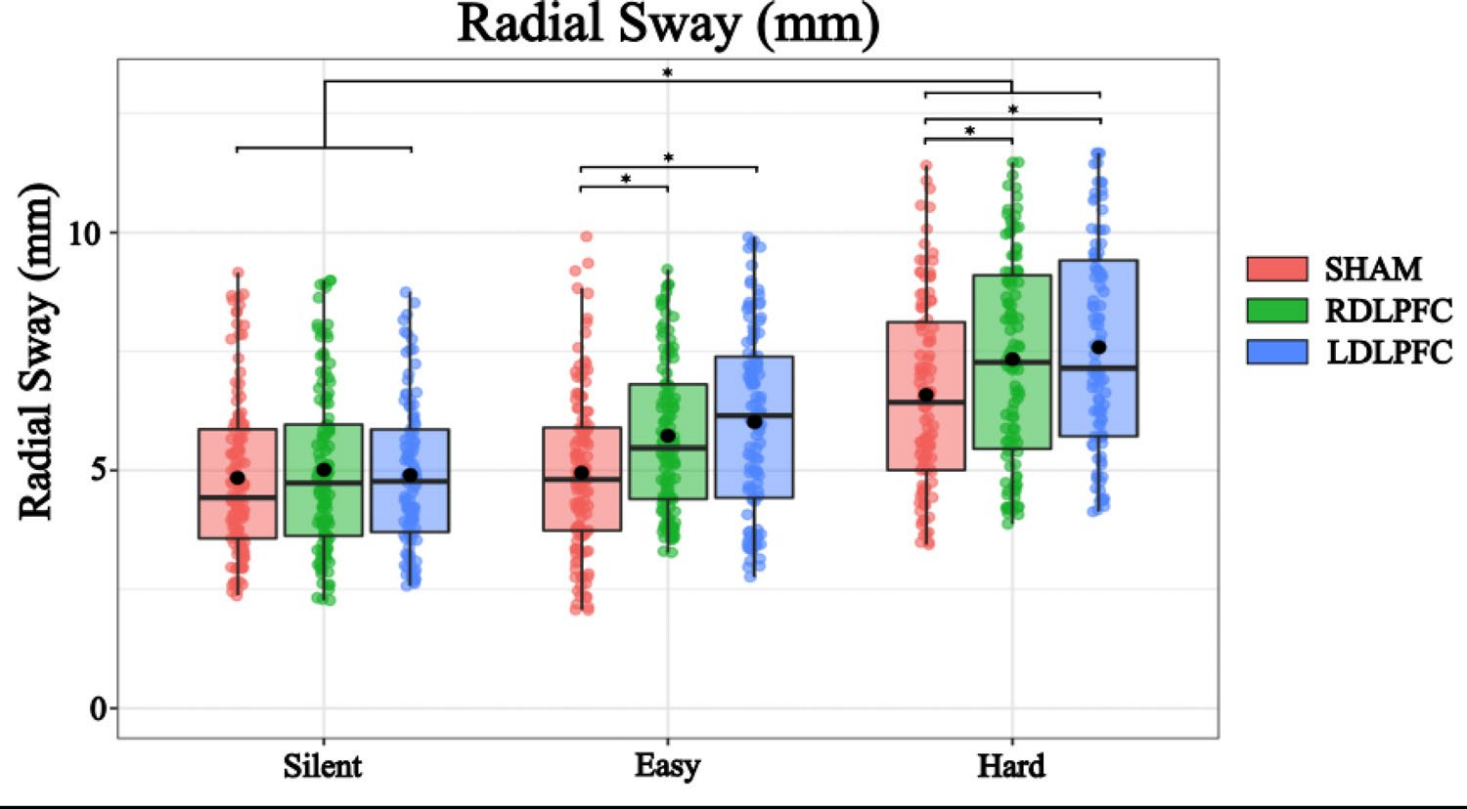
Radial Sway is significantly increased with the introduction of Cognitive Load, with the hard condition causing the strongest effect. During TMS Stimulation, both the left and right stimulation of the DLPFC caused an increase of Radial Sway. Box and whiskers plot with the solid black line representing the median, the solid black dot representing the mean, and the extending lines showing the maximum and minimum values. Each individual dot represents the Radial Sway value of each of the trial across all the subjects

A linear mixed-effects model was conducted to examine the effects of TMS condition (Right DLPFC, Left DLPFC, Sham) and Cognitive Task Difficulty (No task, Easy, Hard) on Radial Sway. The intercept was estimated at 4.921 (SE = 0.217, η = −0.42, t = 22.689) indicating a baseline level of Radial Sway. For TMS condition, stimulation of the Right DLPFC significantly increased Radial Sway compared to the sham condition (Estimate = 0.433, SE = 0.092, η = 0.25, t = 4.662, *p* < 0.001). Similarly, the stimulation of the Left DLPFC also had an even greater effect (Estimate = 0.597, SE = 0.095, η = 29, t = 6.275, *p* < 0.001) (Fig. 3).

Regarding Cognitive Task Difficulty, performing the Easy Task resulted in a significant increase in Radial Sway (Estimate = 0.385, SE = 0.148, η = 0.19, t = 2.603, *p* = 0.009). The Hard task had a more pronounced effect (Estimate = 1.563, SE = 0.259, η = 0.76, t = 6.033, *p* < 0.001). These results indicate that both TMS to the DLPFC and cognitive load influence postural stability, with Left DLPFC stimulation and the Hard task exerting the strongest effects (Fig. 3).

To further investigate the effects of different levels of TMS Stimulation and Cognitive Load on RS, post hoc comparisons were performed. These comparisons compared various combinations of TMS Stimulation and Cognitive Load using Tukey’s method for multiple comparisons with degrees of freedom calculated using the Kenward-Roger method (Table 1). The following significant findings were observed:

**Table 1:** Post-hoc comparisons for Radial Sway

Contrast	Estimate	SE	DF	t.ratio	*p*.value
Silent SHAM – Easy SHAM	−0.385	0.209	26	−1.852	0.6499
Silent SHAM – Hard SHAM	−1.563	0.365	26	−4.284	0.0058
Easy SHAM – Easy RDLPFC	−0.433	0.095	668	−4.581	0.002
Easy SHAM – Easy LDLPFC	−0.598	0.097	688	−6.120	< .001
Hard SHAM – Hard RDLPFC	−0.433	0.094	668	−4.581	0.002
Hard SHAM – Hard LDLPFC	−0.598	0.098	683	−6.120	< 0.001

These results indicate that both TMS Stimulus and Cognitive Condition significantly influence RS, with notable differences between specific levels of these factors. The use of Tukey’s method for multiple comparisons ensures that the reported p-values are adjusted for the family-wise error rate, providing robust statistical inference. (The entire table of post hoc comparisons can be found in the supplementary section; Supplementary Table 1).

### High-frequency radial sway

**Fig. 4:**
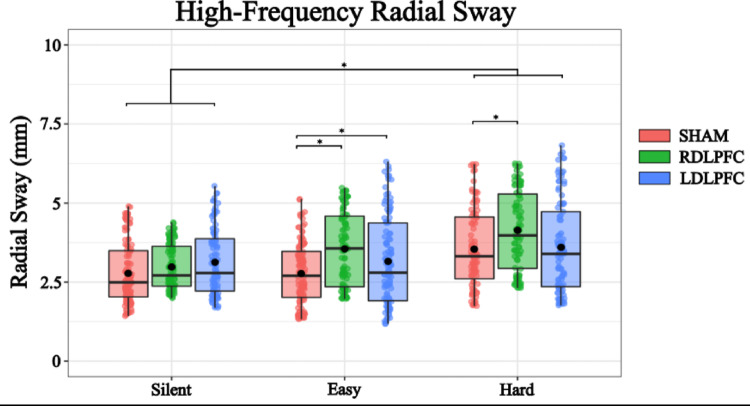
High-Frequency Radial Sway was significantly increased with the introduction of TMS stimulation and the hard condition of the cognitive task. Box and whiskers plot with the solid black line representing the median, the solid black dot representing the mean, and the extending lines showing the maximum and minimum values. Each individual dot represents the Radial Sway value of each of the trial across all the subjects

A linear mixed-effects model was conducted to examine the effects of TMS condition (Right DLPFC, Left DLPFC, Sham) and Cognitive Task Difficulty (No task, Easy, Hard) on Radial Sway. The intercept was estimated at 2.461 (SE = 0.118, η = −0.65, t = 20.833) indicating a baseline level of Radial Sway. For TMS condition, stimulation of the Right DLPFC significantly increased Radial Sway compared to the sham condition (Estimate = 0.414, SE = 0.052, η = 0.34, t = 7.885, *p* < 0.001). Similarly, the stimulation of the Left DLPFC also had a significant effect (Estimate = 0.253, SE = 0.052, η = 0.21, t = 4.898, *p* < 0.001) (Fig. 4).

Regarding the Cognitive Task Difficulty, performing the Easy Task resulted in a small, marginally significant increase in Radial Sway (Estimate = 0.175, SE = 0.091, η = 0.14, t = 1.907, *p* = 0.057). The Hard task had a more pronounced effect (Estimate = 0.532, SE = 0.127, η = 0.43, t = 4.193, *p* < 0.001). These results suggest that both TMS to the DLPFC and increasing cognitive task load affect postural stability, with Right DLPFC stimulation and the Hard task producing the strongest effects (Fig. 4).

To further investigate the effects of different levels of TMS Stimulation and Cognitive Load on RS, post hoc comparisons were performed. These comparisons compared various combinations of TMS Stimulation and Cognitive Load using Tukey’s method for multiple comparisons with degrees of freedom calculated using the Kenward-Roger method (Table 2). The following significant findings were observed:

**Table 2:** Post-hoc comparisons for High-Frequency Radial Sway

Contrast	Estimate	SE	DF	t.ratio	*p*.value
Silent SHAM – Easy SHAM	−0.176	0.131	26	−1.348	0.9070
Silent SHAM – Hard SHAM	−0.532	0.179	26	−2.598	0.1195
Easy SHAM – Easy RDLPFC	−0.414	0.054	622	−7.596	< 0.001
Easy SHAM – Easy LDLPFC	−0.253	0.053	622	−4.766	0.001
Hard SHAM – Hard RDLPFC	−0.414	0.044	622	−7.589	< 0.001
Hard SHAM – Hard LDLPFC	−0.253	0.053	622	−6.120	0.001

### Low-frequency radial sway

**Fig. 5:**
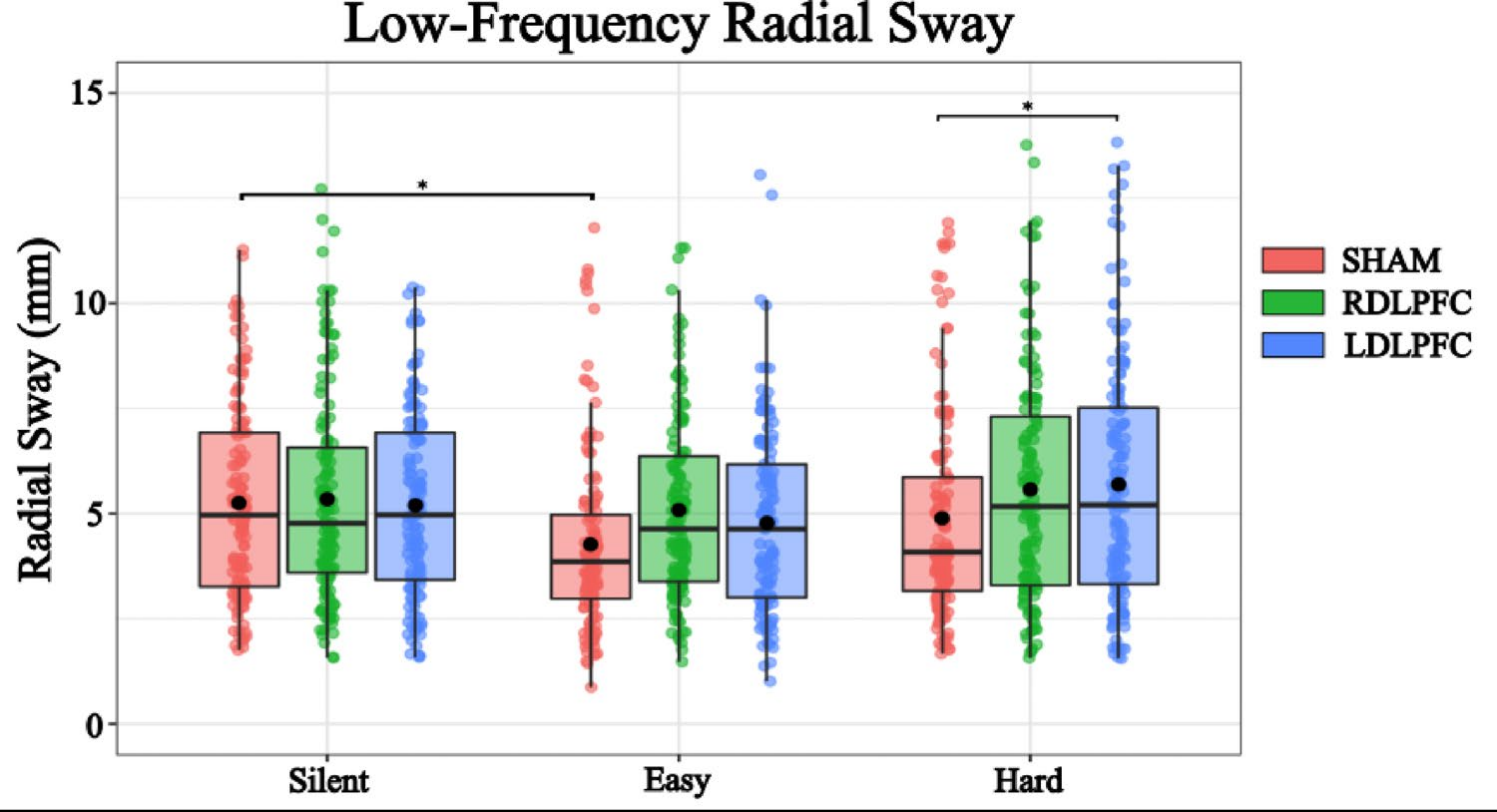
Low-frequency Radial Sway was reduced during the easy difficulty condition of cognitive performance. Similarly, within the cognitive task performance, TMS stimulation caused a significant increase in Low-frequency Radial Sway. Box and whiskers plot with the solid black line representing the median, the solid black dot representing the mean, and the extending lines showing the maximum and minimum values. Each individual dot represents the Radial Sway value of each of the trial across all the subjects

A linear mixed-effects model was conducted to examine the effects of TMS condition (Right DLPFC, Left DLPFC, Sham) and Cognitive Task Difficulty (No task, Easy, Hard) on Radial Sway. The intercept was estimated at 4.787 (SE = 0.206, η = −0.05, t = 23.261) indicating a baseline level of Low Frequency Radial Sway. For TMS conditions, stimulation of the Right DLPFC significantly increased Radial Sway compared to the sham condition (Estimate = 0.531, SE = 0.095, η = 0.29, t = 5.590, *p* < 0.001). Similarly, the stimulation of the Left DLPFC also had a significant effect (Estimate = 0.606, SE = 0.096, η = 0.33, t = 6.297, *p* < 0.001) (Fig. 5).

Regarding the Cognitive Task Difficulty, performing the Easy Task resulted in a small, marginally significant increase in Radial Sway (Estimate = −0.763, SE = 0.117, η = −0.42, t = −6.545, *p* < 0.001). The Hard task had a more pronounced effect (Estimate = -0.536, SE = 0.181, η = −0.29, t = −2.966, *p* = 0.003). These results suggest that both TMS to the DLPFC and increasing cognitive task load affect postural stability, with Right DLPFC stimulation and the Hard task producing the strongest effects (Fig. 5).

To further investigate the effects of different levels of TMS Stimulation and Cognitive Load on RS, post hoc comparisons were performed. These comparisons compared various combinations of TMS Stimulation and Cognitive Load using Tukey’s method for multiple comparisons with degrees of freedom calculated using the Kenward-Roger method (Table 3). The following significant findings were observed:

**Table 3:** Post-hoc comparisons for Low-Frequency Radial Sway

Contrast	Estimate	SE	DF	t.ratio	*p*.value
Silent SHAM – Easy SHAM	0.763	0.165	26	4.621	0.0025
Silent SHAM – Hard SHAM	0.536	0.255	26	2.104	0.4917
Easy SHAM – Easy RDLPFC	−0.531	0.097	622	−5.466	< 0.001
Easy SHAM – Easy LDLPFC	−0.605	0.098	622	−6.152	< 0.001
Hard SHAM – Hard RDLPFC	−0.530	0.097	622	−5.425	< 0.001
Hard SHAM – Hard LDLPFC	−0.601	0.078	622	−5.125	< 0.001

### Detrended fluctuation analysis

A linear mixed-effects model was conducted to examine the effects of transcranial magnetic stimulation (TMS) to the dorsolateral prefrontal cortex (DLPFC) and cognitive task difficulty on postural stability through the calculated alpha value (DFA). Fixed effects in the model included stimulation to the right DLPFC (StimRDLPFC), stimulation to the left DLPFC (StimLDLPFC), and task difficulty conditions (Easy, Hard), with the baseline condition serving as the reference level.

**Fig. 6:**
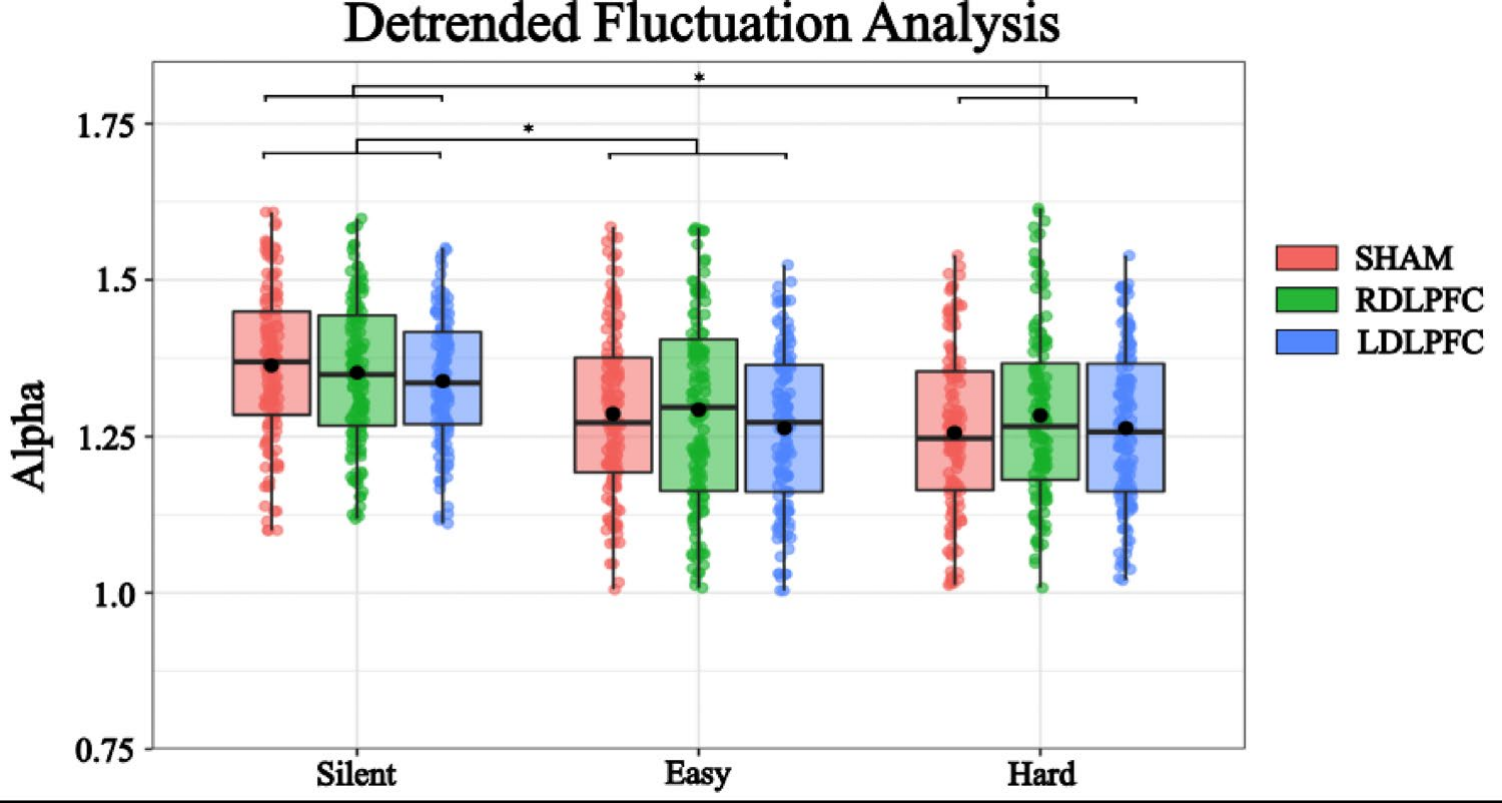
Detrended Fluctuation Analysis: Alpha was reduced during the Easy and Hard condition of cognitive performance. There was no effect of stimulation on DFA. Box and whiskers plot with the solid black line representing the median, the solid black dot representing the mean, and the extending lines showing the maximum and minimum values. Each individual dot represents the Radial Sway value of each of the trial across all the subjects

The model revealed a significant intercept, estimate = 1.36, SE = 0.01, η = 0.49, t = 130.484, *p* < 0.001, indicating the estimated alpha value in the baseline condition. Stimulation to the right DLPFC did not significantly affect alpha value (estimate = 0.005, SE = 0.006, η = 0.05, t = 0.88, *p* = 0.380), nor did stimulation to the left DLPFC (estimate = −0.008, SE = 0.0060, η = −0.07, t = −1.347, *p* = 0.178), suggesting that TMS did not meaningfully alter balance control in this context (Fig. 6).

Conversely, cognitive task difficulty significantly reduced alpha value. Compared to baseline, the Easy condition led to a significant decrease in alpha value (β = -0.071, SE = 0.008, η = −0.62, t = -8.594, *p* < 0.001), and the Hard condition further decreased sway (β = −0.085, SE = 0.008, η = -0.73, t = −10.084, *p* < 0.001). These results indicate that performing cognitive tasks while standing improves postural adaptability, with a stronger effect in the more cognitively demanding condition. However, it should be noted that all values remained within the range expected of alpha (Fig. 6).

To further investigate the effects of different levels of TMS Stimulation and Cognitive Load on RS, post hoc comparisons were performed. These comparisons compared various combinations of TMS Stimulation and Cognitive Load using Tukey’s method for multiple comparisons with degrees of freedom calculated using the Kenward-Roger method (Table 4). The following significant findings were observed:

**Table 4:** Post-hoc comparisons for DFA of Radial Sway

Contrast	Estimate	SE	DF	t.ratio	*p*.value
Silent SHAM – Easy SHAM	0.713	0.012	26	5.967	0.0001
Silent SHAM – Hard SHAM	0.085	0.011	26	7.257	< 0.0001
Easy SHAM – Easy RDLPFC	0.005	0.006	622	−0.860	0.9948
Easy SHAM – Easy LDLPFC	0.008	0.006	622	1.342	0.9242
Hard SHAM – Hard RDLPFC	−0.005	0.006	622	−0.871	0.9948
Hard SHAM – Hard LDLPFC	−0.008	0.006	622	1.545	0.9932

## Discussion

Overall, the results of this study indicate that both cognitive task difficulty and TMS stimulation of the DLPFC influence postural sway dynamics. Specifically, the more challenging the cognitive task, whether through task difficulty itself or TMS downregulation of the frontal cortex, the more significant the increase of the multi-directional variability of CoP becomes, compared to easy or sham conditions. These findings suggest that cognitive processes (through the DLPFC) and postural control (cerebellum and other supra-spinal processes) are in communication with one another, at least when performed concurrently. Reinforcing the idea that maintaining balance may involve executive functioning resources and frontal cortical regions. More directly, we were able to elicit a decrease of postural stability in healthy young adults with the inclusion of a working memory based cognitive task. Furthermore, this effect was amplified based on the degree of cognitive load the task applied, shown through the difficulty of the task as well as the introduction of TMS stimulation to the right and left DLPFC.

Similarly, both the low- and high-frequency mechanisms of sway were modulated by cognitive load through task difficulty and TMS cortical downregulation. The low-frequency components of sway are believed to show the drift of the body’s inertial mass (Winter et al. 1998) and are particularly sensitive to alterations in the sensory environment (Yeh et al. 2010, 2014; van den Heuvel et al. 2009). In contrast, high-frequency components are thought to capture finer corrective adjustments around the center-of-mass, closely related to joint stiffness and muscle activation (Kiemel et al. 2005; van den Heuvel et al. 2009). Our findings indicate that increasing cognitive task difficulty leads to greater variability in both the high- and low-frequency components of radial sway, suggesting a reduction in the stability and adaptability of the postural control system with the inclusion of heightened cognitive load. This pattern emerged not only when task demands increased (i.e., Easy vs. Hard conditions), but also when the downregulation of fronto-cortical regions compromised cognitive performance capabilities.

Following a similar trend as seen in the previous two analyses, DFA was significantly influenced by cognitive load. During cognitive task performance, the DFA scaling coefficient (α) of postural sway decreased, indicating an increase in anti-persistence within the sway parameters when the cognitive load increased. However, this scaling coefficient of postural sway was not impacted by TMS stimulation. Within this context, stability can be conceptualized as the co-adjustment of local variability and serial correlational properties of the CoP. A higher α indicates greater persistence, or a stronger association between successive points of postural sway over time, while a lower α suggests more anti-persistence in sway, or a more tightly regulated system, exhibiting a system that is less resistant to perturbations or changes in the CoP displacement, which in turn reflects the system’s adaptability (Ducharme and van Emmerik 2018). This causes a slight discrepancy within the results, with an increase in radial sway with the heightened cognitive load but a decrease in the DFA value under the same conditions. This result requires further interpretation but may be explained by an increase in the multidirectional variability of CoP contributing to a higher need for control within the fractal properties of DFA.

While working memory performance relies on frontal cortical regions, like the DLPFC, the cerebellum has been implicated in cognitive functioning as well. Both verbal and non-verbal working memory tasks, including the n-back task, have been shown to reliably activate the cerebellum (Chen & Desmond 2005a, 2005b; Tomasi et al. 2005; Beneventi et al. 2007; Hautzel et al. 2009; Stoodley, et al. 2010). This activation pattern implies a strong connection between the primary regions typically associated with cognitive functioning (frontal cortex) and postural control. When cognition is performed while standing, the cerebellum may become activated, alongside the activation patterns necessary for postural sway causing interference between the two tasks. When the frontal regions of interest (DLPFC) are downregulated through TMS, that may cause the regions within the cerebellum to become disrupted as well, causing further interference to the postural control system. Similarly, there have been cortical regions implicated in human balance control. By using functional near-infrared spectroscopic system, Mihara et al. (2008) showed significant activation in the prefrontal cortex, including the dorsolateral prefrontal cortex after a perturbation of the postural control system. This study showed how prefrontal involvement may be relevant to the allocation of attention during postural control, specifically under perturbation and more significantly, this study allows us to better understand the cortical mechanisms for balance control in humans, and possibly, the underlying pathophysiology of falls.

Similarly, previous research has demonstrated that a shift in conscious, attention-driven control of postural stability increases the possibility of disturbing the natural coordination and dynamics exhibited by postural sway (Masters & Maxwell 2008; Wulf, et al. 2001). Often attributed to reinvestment theory (Masters & Maxwell 2008; Masters 1992), this theory posits that automated motor behaviors (such as postural control) become compromised when consciously accessed through task-relevant declarative knowledge (Masters 1992). Additionally, reinvestment theory suggests that aging and neurological diseases lead to an increase in the effect of reinvestment (Masters & Maxwell 2008; Schaefer, et al. 2015a). This was corroborated by Seidler et al. (2010), who discovered that changes associated with aging and injury result in a decline in gray and white matter within the central nervous system (CNS), leading to a reorganization of cortical activation patterns (Ghai, et al. 2017). Based on these findings, the authors proposed that differential activity within the higher neural centers could influence the tasks being performed, potentially increasing conscious attention while performing secondary motor or cognitive tasks (Talelli et al. 2008).

Large-scale motor synergies involved in coordinated actions like standing and walking have been wildy recognized as autonomous systems that support balance, stability, and locomotion (Stoffregen et al. 1999, 2000). Similarly, motor physiology research focused on perception, language, memory, and executive functioning has tended to neglect how motor control dynamics may influence these processes and vice versa (Rosenbaum 2005). Although historically, this interaction has been ignored, current work documenting age-comparative work on posture-cognition (Woollacott & Shumway-Cook 2002) and gait-cognition (Schaefer et al. 2015b) shows that postural control synergies are not as autonomous as previously assumed, and how certain forms of motor behaviors are 'cognitively penetrable' (Teasdale et al. 1993). This recent increase in the corpus of work on cognitive coordination has made it difficult to understand clearly why these systems interact (Fraizer & Mitra 2008).

Dual-task paradigms have gained increasing prevalence in research (Masters & Maxwell 2008; Schaefer et al., 2015a, b; Donker et al. 2007). In cognitive-motor dual-task paradigms, the performer's attention is directed toward an external source of activity (e.g., N-back, mental arithmetic, letter string memorization) concurrently with the execution of a motor task (standing upright). According to the constrained action hypothesis, this attentional change might allow the motor system to function more automatically, resulting in a more effective output (Wulf et al. 2001). However, an increase in the complexity of the behavior has shown a subsequent increase in centralized interference (Lanzarin et al. 2015; Boes et al. 2012; Andrade et al. 2014; Montero-Odasso et al. 2009), which leads to an adverse effect on both the cognitive and motor performance (Boisgontier et al. 2013; Montero-Odasso et al. 2009).

This study demonstrated the reciprocal influence between cognition and postural control. The cognitive task negatively impacted postural stability, with greater task difficulty amplifying this effect. When TMS was administered, it further disrupted participants' cognitive performance across both difficulty levels while simultaneously amplifying the cognitive task's impact on postural stability beyond the effect of difficulty alone. These results further emphasize the frontal cortex’s possible connection to the postural control system, and motor control by showing the impact negatively affecting the frontal cortex has on postural control. These findings highlight the interconnected nature of cognition and postural control, showing that performing both simultaneously impairs performance in both domains. Moreover, it was not just cognition in general that influenced postural control, but specifically working memory and the role of the DLPFC in this interaction.

## Limitations

One limitation of the present study is the prioritization of visually observed muscle twitches to determine active motor threshold (AMT), despite the availability of EMG recordings. Visually determined AMTs are known to be higher than those based on EMG due to the need for more overt muscle activation, which may lead to overestimation of the true threshold. This, in turn, could result in higher-than-intended stimulation intensities, potentially producing broader and less focal neuromodulatory effects that may extend to adjacent cortical regions, such as the primary motor cortex. While this approach was consistent with prior protocols, future studies would benefit from using EMG-based thresholds to enhance objectivity, reproducibility, and focality of stimulation.

Another limitation concerns the calculation of radial sway (RS) using absolute center of pressure (CoP) coordinates without centering on the mean CoP position, following the method outlined by Lafond et al. (2004). Although participants’ foot positions were standardized, it remains unclear whether mean CoP position remained stable across all experimental conditions. Factors such as TMS administration or concurrent cognitive tasks may have altered participants’ postural alignment or weight distribution, potentially introducing global shifts in posture. As a result, RS values may partially reflect these shifts rather than true sway magnitude, complicating the interpretation of RS as a direct measure of postural stability. Future work may benefit from computing RS relative to the mean CoP position within each trial to more accurately capture sway-related variability, particularly in contexts where systematic postural changes are expected.

## Data Availability

Datasets generated and analyzed during the current study are available from the corresponding author upon reasonable request.
